# Perfluorodecanoic acid stimulates NLRP3 inflammasome assembly in gastric cells

**DOI:** 10.1038/srep45468

**Published:** 2017-04-03

**Authors:** Xiangyu Zhou, Tianyi Dong, Ziyan Fan, Yanping Peng, Rongbin Zhou, Xiaqiong Wang, Ning Song, Mingyong Han, Bingbing Fan, Jihui Jia, Shili Liu

**Affiliations:** 1 Department of medical microbiology, School of basic medical science, Shandong University, Jinan, Shandong, 250012, China; 2 Shandong Provincial Hospital, Shandong University, Jinan, Shandong, 250021, China; 3 China National Tobacco Quality Supervision & Test Center, 2 Fengyang Street, Zhengzhou, Henan, 450001, China; 4 Institute of Immunology and CAS Key Laboratory of Innate Immunity and Chronic Disease, School of Life Sciences and Medical Center, University of Science and Technology of China, Hefei, Anhui, 230027, China; 5 School of Public Health, Shandong University, Jinan, Shandong, 250012, China

## Abstract

Perfluorodecanoic acid (PFDA), a perfluorinated carboxylic acid, presents in the environment and accumulates in human blood and organs, but its association with tumor promotion are not clear. Given that inflammation plays a significant role in the development of gastric malignancies, we evaluated the effects of PFDA on activation of the inflammasome and inflammation regulation in the gastric cell line AGS. When added to cell cultures, PFDA significantly stimulated IL-1β and IL18 secretion and their mRNA levels compared with control cells. By RT-PCR and western-blot we found that up-regulation of NLRP3 were associated with promotion of IL-1β and IL-18 production. Then expression variation of cIAP1/2, c-Rel and p52 were analyzed, the results demonstrated raised mRNA expression in all the tested genes concomitant with enhanced inflammasome activity after exposure to PFDA. Assays with cIAP2 siRNA and NFκB reporter provided additional evidence that these genes were involved in PFDA-induced inflammasome assembly. Furthermore, increased secretion of IL-1β and IL-18 were detected in stomach of PFDA-treated mice, disorganized alignment of epithelial cells and inflammatory cell infiltration were also observed in the stomach tissues upon PFDA treatment. This study reports for the first time that PFDA regulates inflammasome assembly in human cells and mice tissues.

Gastric cancer is the second leading cause of cancer death worldwide and accounts for about 10% of all invasive cancers^1^. Although the pathogenesis of gastric cancer is not completely understood, it is worth noting that a positive correlation of gastric cancer with environmental pollution has been confirmed^2^,^3^,^4^. As an illustration, the total number of cases and deaths from gastric cancer have increased concomitant with extensive demographic changes and ongoing increase of environmental pollution in China.

Perfluorodecanoicacid (PFDA) is a highly toxic perfluorinated fatty acid (PFCA)^5^, a kind of persistent environmental pollutant. PFDA is present in air, food, and water, especially in China, where 139ng/L PFDA was detected in snow fall in the area around Beijing^6^. PFDA intake is primarily from foods and drinking water^7^,^8^, and it can accumulate in human blood and organs. Moreover, its serum elimination half-life lasts several years. PFDA has been reported to induce hypophagia and severe weight loss, bradycardia, hypothermia, and decreased serum thyroid hormone levels in rats^9^,^10^. So far, there is certainly a need for more data in the field of PFDA-induced cancers.

While the pathogenesis of gastric cancer is not completely understood, epidemiological studies suggest that chronic inflammation plays a significant role in the development of gastric malignancies^11^. Emerging evidence indicates that the inflammasome is an essential component of the innate immune system, playing a central role in regulating inflammation^12^. The inflammasome complex is composed of a nucleotide binding domain, leucine-rich repeat-containing receptor (NLR), caspase-1 and the ASC (apoptosis-associated speck-like protein containing a caspase recruitment domain) adaptor protein, which is necessary to join NLR and caspase-1^13^. The activation of the inflammasome complex triggers the recruitment and activation of caspase-1, which is necessary for the maturation and release of two important proinflammatory cytokines, IL-1β and IL-18^13^. Different inflammasomes have been described, i.e. NLRP1, NLRP3 and NLRC4. Inflammasomes assembly generally requires NFκB activation and danger signals, generating active caspase-1 that cleaves the precursor forms of IL-1β and IL-18 into the mature and active forms. Some evidence suggested that NLRs are closely correlated to cancer occurrence, the level of IL-1β and IL-18 were found to be significantly elevated in various types of malignancies^12^,^14^,^15^. Therefore, elucidating the mechanisms that involve activation of the inflammasomes and their modulatory effects has become a novel strategy for inflammation regulation and cancer prevention research.

In this study, we found that PFDA induced IL-1β and IL-18 secretion in culture solution of cell line AGS and mice stomachs. Our results demonstrated that the IL-1β and IL-18 inductions were associated with activation of NLRP3 inflammasome. This study reports for the first time that PFDA activates inflammasome and promotes gastric inflammation in gastric cells and mice tissues.

## Material & methods

### Cell line and animal treatment

Human gastric adenocarcinoma cell line AGS, huaman hepatocellular carcinoma cell lines BEL7402 and human leukemia macrophage cell line THP-1 were all from American type culture collection (ATCC) and maintained in our laboratory. They were cultured in Ham’s F-12 medium (HyClone, Utah, USA) containing 10% FCS and 1% penicillin-streptomycin. PFDA was purchased from Sigma Chemical Company (CAS number 335-76-2, St. Louis, MO, USA). Different volumes of PFDA were added into cell culture media for treatments. Six-week-old female Balb/c mice were acclimated and caged in groups of 5 or less. NLRP3 knockout mice were kindly offered by Professor Rongbin Zhou (University of Science and Technology of China). Mice were administered with PFDA at a dose of 25 mg/kg/d in drinking water, 0.014 M dimethyl sulfoxide (DMSO) was utilized as the control. Mice treatment and dissection were performed according to the protocol approved by the Ethics Committee on Animal Experiments of Medical School of Shandong University. Stomachs were harvested 18d after PFDA dosing. Some stomachs were cut into small pieces and mixed with Phosphate-buffered saline in a certain percentage for tissue grinding. The obtained tissue homogenates were used for subsequent Enzyme-linked immunosorbent assay. Some stomachs were Formalin-fixed and paraffin-embedded for Hematoxylin and eosin staining. The rest of stomachs were snap-frozen in liquid nitrogen and stored at −80 °C for RNA extraction and quantitative RT-PCR. FuGENEs HD Transfection Reagent (Roche Applied Science, Basel, Switzerland) was used for transfection. All transfections were performed according to the manufacturer’s instructions.

### RNA extraction and quantitative real-time PCR

Total cellular RNA was extracted with Trizol (Life Technologies, California, USA) according to the protocol provided by the manufacturer. First-strand cDNA was synthesized from 1 μg total cellular or tissue RNA using the RevertAid TM First Strand cDNA Synthesis Kit (Thermo Fisher Scientific, Massachusetts, USA) with random primers. Then cDNA was amplified for quantitative real-time PCR, the specific primers used were as follows: for human cIAP1(Birc2), forward primer 5′-AATGGAAGATAGCACGAT-3′ and reverse primer 5′-CCTTTCTGAGACAGGCAC-3′; cIAP2(Birc3), forward primer 5′-GCCTGATGCTGGATAACT-3′ and reverse primer 5′-GAATAAGAGCCACGGAAA-3′; for human NLRP3, forward primer 5′-TGAACAGCCACCTCACTT-3′ and reverse primer 5′-CAACCACAATCTCCGAAT-3′; for human NLRP1, forward primer 5′-CCAGTTTGTGCGAATCCA-3′ and reverse primer 5′-CCAACGTAGAACTCCGAGAA-3′; for human NLRC4, forward primer 5′-CAATAGCCGAGCCCTTAT-3′ and reverse primer 5′-AGCCAAATCGTCCAAGTC-3′; for human IL-1B, forward primer 5′-ACAGTGGCAATGAGGATG-3′ and reverse primer 5′-TGTAGTGGTGGTCGGAGA-3′; human IL-18, forward primer 5′-ATAGCCAGCCTAGAGGTA-3′ and reverse primer 5′-ATCAGGAGGATTCATTTC-3′; for β-actin, forward primer 5′-AGTTGCGTTACACCCTTTCTTG-3′ and reverse primer 5′-CACCTTCACCGTTCCAGTTTT-3′. The real-time PCR reactions were performed at: 95 °C, 10 sec (denaturation); 55 °C, 30 sec (annealing); 72 °C, 30 sec (extension) for 35 cycles. The real-time PCR reactions were performed on the ABI7000 Fast Real-Time PCR System with SYBR Premix Ex Taq TM according to the procedures.

### Enzyme-linked immunosorbent assay (ELISA)

IL-1β and IL-18 in culture solution or mouse gastric tissues were detected with human/mouse IL-1β and IL-18 ELISA Kit (4 A Biotech Co., Ltd, Beijing, China) according to the manufacturer’s instructions. Mice treatment and dissection were performed according to the protocol approved by the Ethics Committee on Animal Experiments of Medical School of Shandong University. Briefly, add 100 μl diluted Standard in triplicate or 100 μl sample to each well, then add 50 μl biotin labeled Antibody Working Solution per well. After aspiration and wash for 4 times, incubate for 2 hours at room temperature. Next, add 100 μl Enzyme Binding Working Solution to each well, aspirate and wash 4 times. And then add 100 μl Substrate Solution to each well. Protect form light, incubate for 10–20 minutes at RT. Finally, add 100 μl Stop Solution to each well and read at 450 nm within 30 minutes.

### Western Blot Analysis

Western Blot Analysis was performed as described previously^16^. Briefly, cell lysates (20 μg/lane) were separated on 10% SDS polyacrylamide gel and then were transferred to a poly (vinylidene fluoride) membrane. NLRP3 (Cat No.TA336883), cIAP1 (sc-271419) and 2 (Cat No.TA800041), c-Rel (Cat No.TA324346) and p52 (sc-7386) protein was detected by a mouse monoclonal IgG (OriGene Co. Ltd, Beijing, China and Santa Cruz Biotechnology Inc., USA) and visualized by the enhanced chemiluminescence system (Amersham, Arlington Heights, IL). The density of the bands was quantitated using the NIH image software package. The intensity of gene expression was judged by the ratio of their expression in PFDA treated groups to their corresponding expression in DMSO groups, and a ratio of more than 1.0 was considered to be an indication of over-expression.

### siRNA interference

Chemical modified Stealth siRNA targeting cIAP2 and control siRNA were from RiboBio Co., Ltd. (Guangzhou, Guangdong, China). The sequence for cIAP2 siRNA was 5′-GGAGTTCATCCGTCAAGTT-3′. Cells were transfected with siRNA by the Lipofectamine 2000 method (Life Technologies, California, USA).

### Hematoxylin and eosin (H&E) staining

Hematoxylin (Code No. ZLI9606), eosin (Code No. ZLI9612), and diaminobenzidine (DAB, Code No. ZLI9632) staining kits were purchased from Zhongshan Golden Bridge Biotechnology (Beijing, China). Mice treatment and dissection were performed according to the protocol approved by the Ethics Committee on Animal Experiments of Medical School of Shandong University. Formalin-fixed, paraffin-embedded mice stomach tissues were cut into 4-μm thick tissue sections and then stained with hematoxylin and eosin (HE) according to the manufacturer’s instructions. The staining images were acquired using a light microscope to observe the pathological alterations of the stomach tissues.

### Statistical Data analysis

Data were expressed as mean ± standard deviation (SD). Differences between three groups were compared using the Student’s t-tests and ANOVA. All experiments were repeated at least three times and P < 0.05 (*) was considered statistically significant.

## Results

### PFDA exposure increases the secretion and mRNA levels of IL-1β and IL-18 in AGS cells

**Figure 1:**
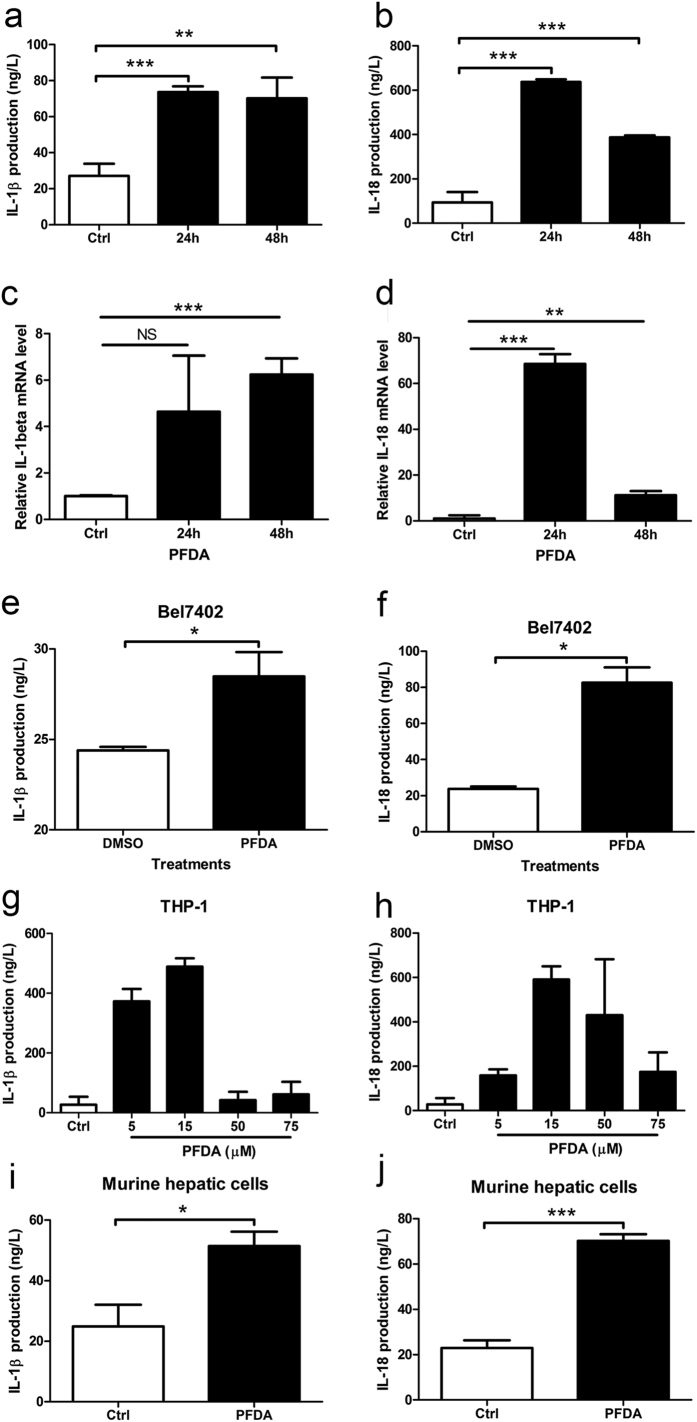
PFDA significantly stimulated IL-1β and IL-18 production. IL-1β and IL-18 secretions in culture media of AGS were increased after 5 μM PFDA treatments (**a** and **b**), ANOVA = 0.011 and <0.0001 respectively); this increased secretion was verified by RT-qPCR (**c** and **d**); IL-1β and IL-18 promotion was also detected in culture media of BEL7402 (**e** and **f**), THP-1 (**g** and **h**), ANOVA = 0.0005 and 0.01058 respectively) and normal murine hepatic cells (**i** and **j**). ELISA and RT-qPCR were performed as described in Materials and Methods.

To assess effects of PFDA on the secretion of the cytokines that may be regulated by the inflammasome mechanism, we treated the gastric epithelial cell line AGS with PFDA and monitored IL-1β and IL-18 productions by enzyme-linked immunosorbent assay (ELISA). As shown in Fig. 1a and b, cells incubated with PFDA had significantly promoted IL-1β and IL-18 secretion in culture media compared with DMSO-treated control cells. PFDA enhanced IL-1β production in culture media by more than 90% compared with control cells within 24 h; this ratio increased to 220% for IL-18. This increased yield of IL-1β and IL-18 was verified by RT-qPCR of AGS cells (Fig. 1c and d). The results confirm that PFDA increased IL-1β and IL-18 production in human gastric cells.

Moreover, the human hepatic cell line Bel7402, macrophage cell line THP-1 and normal murine hepatic cells were also used for testing IL-1β and IL-18 production by ELISA after treatment with PFDA. As demonstrated in Fig. 1e,f,g,h,i and j, Bel7402, THP-1 and normal murine hepatic cells treated with PFDA raised IL-1β and IL-18 production compared with DMSO-treated control cells. These results support the hypothesis that PFDA has an effect on IL-1β and IL-18 production in human cells.

### PFDA promotes caspase-1 activation in gastric epithelial cells via NLRP3 inflammasome

**Figure 2:**
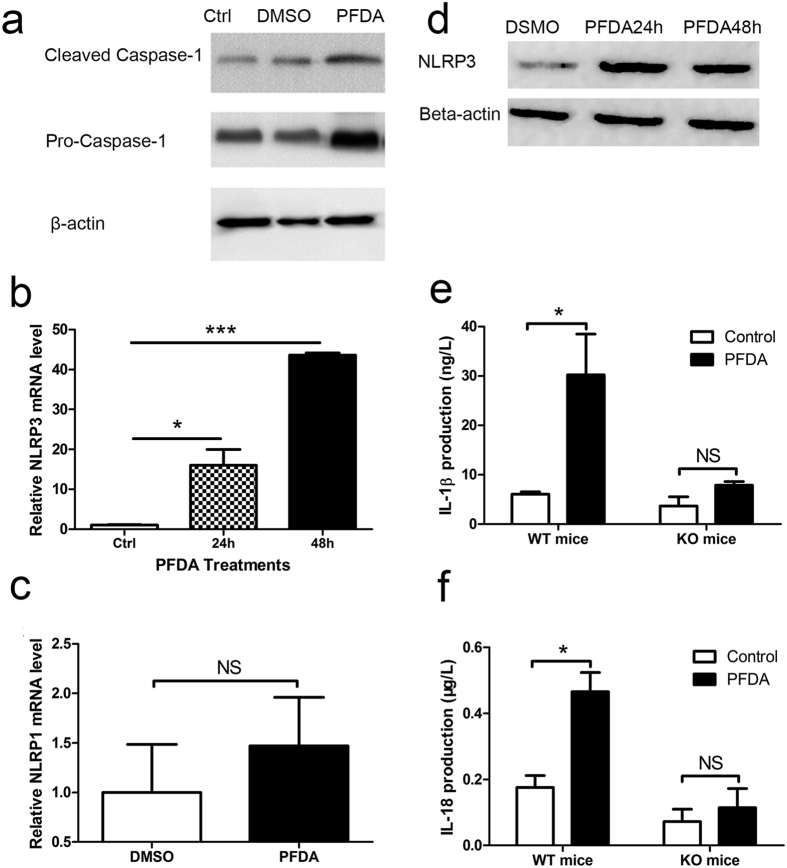
PFDA significantly enhanced NLRP3 inflammasome assembly. (**a**) Caspase-1 expression was increased after 5 μM PFDA treatment for 24 h or 48 h in comparison with DMSO control; (**b**) the mRNA level of NLRP3 was increased by PFDA treatment and in a time-dependent manner (ANOVA <0.0001); On the contrary, NLRP1 had no significant difference in mRNA level in response to PFDA treatments (**c**); This mRNA increase of NLRP3 level upon PFDA exposure was verified by Western blot (**d**). The promotions in IL-1β and IL-18 production were abrogated in their stomach of KO mice (**e** and **f**). RT-qPCR and Western blot were performed as described in Materials and Methods.

To determine if PFDA-promoted secretion of IL-1β and IL-18 was a consequence of increased inflammasome activity, the level of active caspase-1 was examined in AGS concomitant with PFDA treatment. As shown in Fig. 2a, PFDA stimulated production of activate caspase-1.

To find out which inflammasome(s) were involved in PFDA’s action, we performed real-time PCR to analyze the changes of mRNA levels of NLRP1, NLRP3 and NLRC4. The mRNA level of NLRP3 was increased by PFDA treatment and in a time-dependent manner (Fig. 2b). On the contrary, NLRP1 had no significant change in mRNA level in response to PFDA treatments (Fig. 2c), whereas NLRC4 could not be detected (no visible peak). This observation was consistent with an increase of NLRP3 protein level upon PFDA exposure as detected by Western blot (Fig. 2d). The dependence of cytokine secretion on NLRP3 was further verified by NLRP3 knockout mice, the promotions in IL-1β and IL-18 production were abrogated in their stomach (Fig. 2e and f).

### cIAP1 and -2, which are required for efficient caspase-1 activation by the inflammasomes, were involved in PFDA induced NLRP3 inflammasome activation

**Figure 3:**
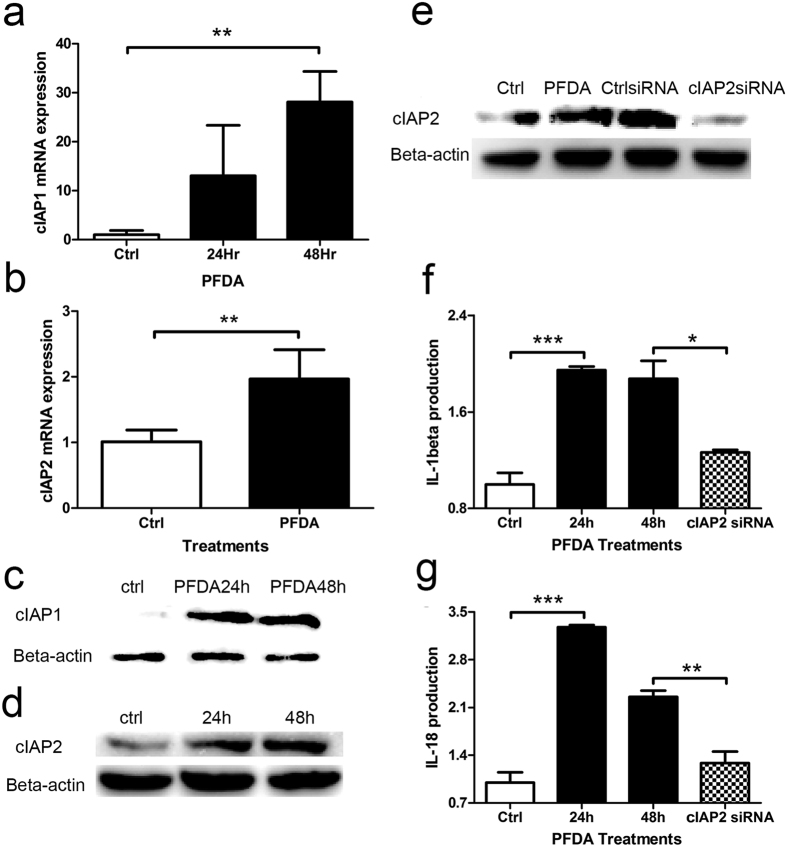
cIAP1 and -2 were involved in PFDA-associated NLRP3 inflammasome assembly promotion. (**a** and **b**), cIAP1 and -2 were activated by PFDA at mRNA level, this was evidenced by western blot at protein level (Fig. 3c and d). (**e**), inhibition of cIAP2 by siRNA abrogated PFDA-induced cIAP2 production; (**f** and **g**), inhibition of cIAP2 reduced IL-1β and IL-18 secretion. ELISA, Western blot and RT-qPCR were performed as described in Materials and Methods.

cIAP1 and cIAP2 are critical effectors of the inflammasomes and are required for inflammasome assembly and Caspase-1 activation. siRNA-mediated depletion of endogenous cIAP1 or cIAP2, alone or in combination, dramatically blunted caspase-1 activity^17^. Therefore, we thought to determine if cIAP1 and cIAP2 were involved in PFDA-promoted inflammasome activation. As shown in Fig. 3a and b, cIAP1 and cIAP2 mRNA levels were increased by PFDA, as shown by western blot at protein level (Fig. 3c and d). To confirm the role of cIAPs in PFDA-induced inflammasome activation, we investigated the effect of inhibition of cIAP2, by cIAP2 siRNA, on activation of caspase-1 and secretion of IL-1β and IL-18. As shown in Fig. 3e,f and g, inhibition of cIAP2 abrogated PFDA-induced cIAP2 production and IL-1β/IL-18 secretion.

### NFκB activity was enhanced in PFDA-treated gastric epithelial cells

**Figure 4:**
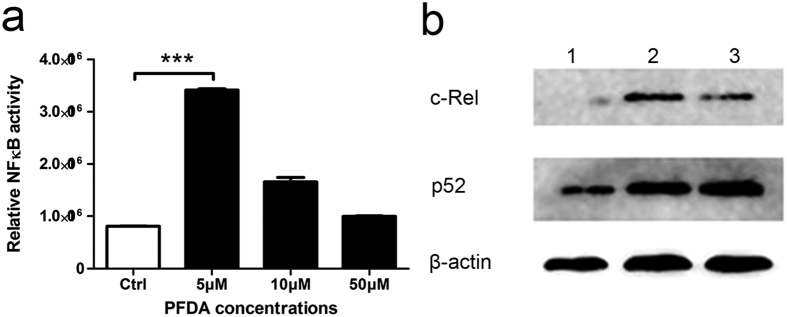
PFDA significantly enhanced NFκB activity. (**a**) NFκB activity was increased after PFDA treatment for 24 h in comparison with DMSO control and in a dose-dependent manner in response to PFDA, ANOVA <0.0001; (**b**) This activation of NFκB upon PFDA exposure was verified by the expression of c-Rel and p52: 1, DMSO control; 2, AGS with PFDA treatment for 24 h; 3, AGS with PFDA treatment for 48 h. NFκB reporter assay was performed as described in the Kit instruction manual and Western blot were performed as described in Materials and Methods.

NFκB plays an important role in activating the priming step of inflammasomes and NFκB inhibition results in a significant reduction of NLRP3 expression. To assess effects of PFDA on NFκB activation, we treated AGS gastric epithelial cells with PFDA and monitored NFκB activity with NFκB reporter plasmid and Duo-Luciferase Assay Kit. As shown in Fig. 4a, cells incubated with PFDA had significantly increased NFκB activity by 240% after PFDA treatment compared with DMSO-treated control cells. The enhanced NFκB activity was verified by enhanced expression of c-Rel and p52 (Fig. 4b).

### Promotion in secretion of IL-1β and IL-18 and inflammation were detected in the stomach of PFDA-treated mice

**Figure 5:**
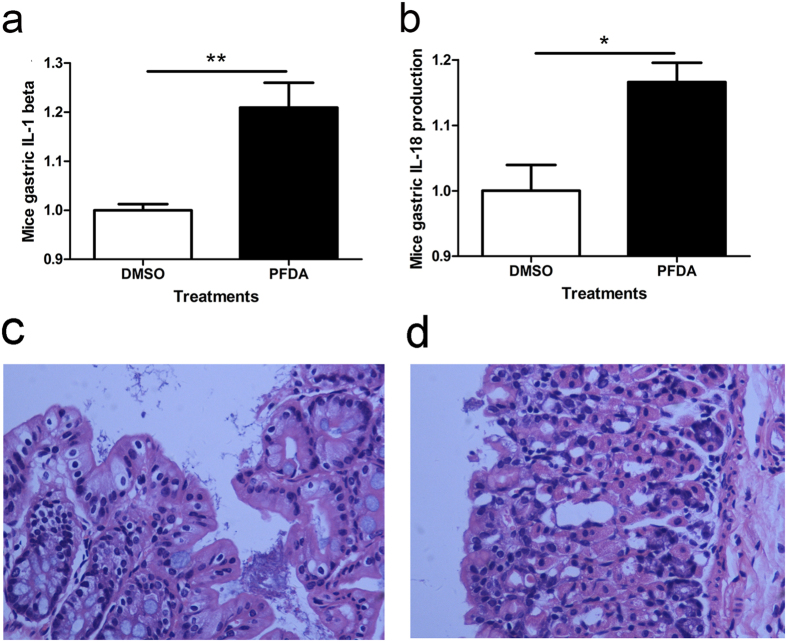
Promotion in secretion of IL-1β and IL-18 and inflammation were detected in the stomach of PFDA-treated mice. IL-1β and IL-18 secretions in mice gastric tissues were increased after PFDA treatments for 18d at 25 mg/kg/d (**a** and **b**); Hematoxylin and Eosin (HE) staining of the stomach tissues of PFDA treated C57BL/6 mice showed disorganized alignment of cells and increased inflammatory cells infiltration (**c** and **d**). ELISA and HE staining were performed as described in Materials and Methods.

To investigate the effects of PFDA on inflammasome activation and inflammation *in vivo*, we treated wild type C57BL/6 mice with PFDA in drinking water for 2 weeks and tested mice stomachs with ELISA. As shown in Fig. 5a and b, IL-1β and IL-18 were increased in the stomach tissues of PFDA treated mice. Moreover, hematoxylin and eosin (HE) staining of stomach tissues of PFDA- or DMSO- treated C57BL/6 mice, showed disorganized alignment of cells and increased inflammatory cells infiltration (Fig. 5c and d). Therefore, these results demonstrate for the first time that PFDA regulates inflammasome assembly and inflammation in human cells and mice tissues.

## Discussion

Perfluorinated carboxylic acids or perfluorinated fatty acids (PFCAs) have been used for decades to make products that resist heat, oil, and water. Because they are used in the manufacture of nonstick cookware, fire-fighting foam, and many other industrial products^18^,^19^, perfluorinated compounds can be detected globally in the environment^6^,^20^, wildlife^21^,^22^,^23^,^24^ and humans^25^,^26^,^27^,^28^,^29^,^30^. Continued monitoring of PFCAs is necessary since these chemicals persist in the environment and accumulate at much higher concentrations in human blood and organs because of their nondegradable nature and extremely long biological half-lives^31^,^32^.

The reported cellular and physiological effects of PFCAs are associated with almost all systems of the human body; reports on the immunotoxicity of PFCs in humans are limited and controversial. On one hand, some reports suggested that exposure to PFCAs might be associated with immunosuppressive effects. Brieger *et al*. reported that Perfluorooctanoic Acid (PFOA) and Perfluorooctane Sulfonate (PFOS) were associated with reduced Natural killer (NK) cell activity and reduced the release of the pro-inflammatory cytokine Tumor necrosis factor (TNF)-α following lipopolysaccharide (LPS)-stimulation^33^. Two recently published studies indicate that PFCAs may lower vaccine protection in children^34^,^35^. Dong *et al*. and Zheng *et al*. discovered that the cytokine balance favored helper T cell (Th)2 responses in experimental C57BL/6 male mice with short-term or subchronic PFOS exposure^36^,^37^. On the other hand, some studies demonstrated that PFCAs act as an immunity enhancer. Wang *et al*. reported that prenatal PFOA and PFOS exposures positively correlated with cord blood IgE levels in boys^38^. In agreement, Emmett *et al*. reported a slight increase in absolute monocyte counts of residents who lived in a water district contaminated with PFOA^39^. Moreover, Ryu *et al*. found that utero-through-adulthood exposure of allergen-naïve mice to PFOA alone induced lung inflammation characterized by macrophage accumulation and IFN-expression, as well as Arylhydrocarbon Receptor (AHR), a hallmark symptom of asthma^40^.

Despite recent advances in understanding the molecular mechanisms of perfluorinated environmental pollutants, many unanswered questions remain. PFDA is a straight chain ten-carbon PFCA that is structurally similar to fatty acids. *In vivo*, PFDA has highly potent and persistent toxicity^5^; it is several times as toxic as PFOA, so PFDA may be important for understanding the molecular mechanisms of toxicity. It is thus hoped that PFDA can assist in dissecting the sequence of events that ultimately results in tumor formation^5^. However, despite the evidence of PFDA toxicity, so far little is known of its effect on malignancy promotion and how it acts in inflammation. In this study, we evaluated the effects of PFDA on activation of the inflammasomes and inflammation regulation in the gastric cell line AGS. When added to cell cultures, PFDA significantly increased NLRP3 inflammasome activation compared with control cells. Moreover, the promotion in synthesis of IL-1β and IL-18 were detected in the stomach of PFDA-treated mice. This report describes for the first time that PFDA regulates inflammasome assembly in gastric cells.

Proteins of the inhibitor of apoptosis protein (IAP) gene family have emerged as among the most important intrinsic inhibitors of apoptosis and could exert important roles in inflammation and innate immunity. They are characterized by the presence of a baculovirus IAP repeat (BIR) domain in one to three copies. cIAP1 and -2 function as ubiquitin ligases^41^,^42^ and were recently identified as important effectors of innate immunity, mediating critical steps in Nucleotide-binding oligomerization domain-containing protein (NOD) signaling^43^. Moreover, cIAP1 and -2 were shown to mediate Mitogen-activated protein kinases (MAPK) activation downstream of Toll-like receptor 4 (TLR4) signaling by catalyzing degradative K48-linked ubiquitination of TNF Receptor Associated Factor 3 (TRAF3)^44^. cIAP2-deficient mice have reduced cytokine amounts in response to LPS and are resistant to LPS endotoxemia^45^. Labbe *et al*. reported that the cIAPs were critical effectors of the inflammasomes and were required for efficient caspase-1 activation. cIAP1, cIAP2, and the adaptor protein TRAF2 interacted with caspase-1-containing complexes and mediated the activating nondegradative K63-linked polyubiquitination of caspase-1^17^. Dagenais *et al*. reported that cIAP2-deficient mice had impaired activation of the regenerative inflammasome–interleukin-18 (IL-18) pathway^46^. Moreover, the results of Mayer *et al*. revealed IAP antagonism as a profound anti-inflammatory principle *in vivo* and highlighted IAPs as important regulators of inflammatory processes in endothelial cells^47^. In this study, we discovered that PFDA activated NLRP3 inflammasome through cIAP1/2, as a result of knockdown with cIAP2 siRNA, providing additional evidence that cIAP1/2 was involved in PFDA-induced inflammasome assembly.

NFκB transcription factors play an important role in many physiological processes and diseases^48^,^49^. IKKβ preferentially phosphorylates the IκB proteins, resulting in the release and activation of canonical NFκB p50/p65 (RelA) and c-Rel/p65 complexes. IKKα homodimer-mediated phosphorylation of cytoplasmic NFκB2/p100 results in partial proteasomal processing of p100 to produce mature p52, thereby activating the alternative pathway^50^. NFκB is thought to serve as the first signal that primes NLR and pro-IL-1β expression^51^,^52^,^53^. Qiao *et al*. demonstrated that NLRP3 promoter contains a putative NFκB binding site and NFκB inhibition resulted in a significant reduction of NLRP3 expression^54^. Meanwhile, mounting evidence suggests that NLRP3 inflammasome formation is positively associated with NFκB activity^55^,^56^. All these findings implied that the pro-tumorigenic ability of NFκB might be attributed to inflammasome activation.

cIAP1 and -2 are required for NFκB signaling. Bertrand *et al*. revealed that cIAP1 and -2 were responsible for K63-linked polyubiquitination of receptor interacting protein 1 (RIP1), promoting NFκB activation signaling^57^. cIAP1 and cIAP2 also direct the ubiquitination of NFκB-inducing kinase (NIK) to maintain the noncanonical NFκB pathway^58^,^59^. In this study, we discovered that PFDA promoted NFκB reporter activity as well as enhanced c-Rel and p52 expression; thereby confirming activation of NFκB signaling. Conclusively, this report describes for the first time that PFDA regulates inflammasome activation and inflammation in gastric cells.

## Additional Information

**How to cite this article:** Zhou, X. *et al*. Perfluorodecanoic acid stimulates NLRP3 inflammasome assembly in gastric cells. *Sci. Rep.* **7**, 45468; doi: 10.1038/srep45468 (2017).

**Publisher's note:** Springer Nature remains neutral with regard to jurisdictional claims in published maps and institutional affiliations.

## References

[b1] Ferlay J. . Estimates of worldwide burden of cancer in 2008: GLOBOCAN 2008. Int J Cancer. 127, 2893–917 (2010). 21351269 10.1002/ijc.25516

[b2] Ashley D. J. Environmental factors in the aetiology of gastric cancer. British journal of preventive & social medicine. 23, 187–189 (1969). 5798461 10.1136/jech.23.3.187 PMC1059194

[b3] Winkelstein W. Jr. & Kantor S. Stomach cancer. Positive association with suspended particulate air pollution. Archives of environmental health. 18, 544–547 (1969). 5773988 10.1080/00039896.1969.10665450

[b4] Zou X. Environmental Pollution and Epidemic of Common Cancers in China. Ke Ji Dao Bao. 32, 58–64 (2014).

[b5] Heuvel J. P. V. Perfluorodecanoic Acid as a Useful Pharmacologic Tool for the Study of Peroxisome Proliferation. Gen. Pharmac. 27, 1123–1129 (1996). 10.1016/0306-3623(95)00126-3 8981056

[b6] Wang J., YY P., YL S. & YQ C. Perfluorinated compounds pollution levels in snowfall of Beijing urban area. Scientia Sinica Chimica. 41, 900–906 (2011).

[b7] D’Hollander W., Voogt P., Coen W. & Bervoets L. Perfluorinated substances in human food and other sources of human exposure. In Reviews of Environmental Contamination and Toxicology. New York, NY: Springer. **p**, 179–215 (2010). 10.1007/978-1-4419-6880-7_4 20811865

[b8] Tittlemier S. . Dietary exposure of Canadians to perfluorinated carboxylates and perfluorooctane sulfonate via consumption of meat, fish, fast foods, and food items prepared in their packaging. J Agric Food Chem. 55, 3203–3210 (2007). 17381114 10.1021/jf0634045

[b9] Langley A. E. & Pilcher G. D. Thyroid, bradycardic and hypothermic effects of perfluoro-n-decanoic acid in rats. Journal of toxicology and environmental health. 15, 485–491 (1985). 4032495 10.1080/15287398509530675

[b10] Olson C. T. & Andersen M. E. The acute toxicity of perfluorooctanoic and perfluorodecanoic acids in male rats and effects on tissue fatty acids. Toxicology and applied pharmacology. 70, 362–372 (1983). 6636169 10.1016/0041-008x(83)90154-0

[b11] Fukata M. & Abreu M. T. Role of Toll-like receptors in gastrointestinal malignancies. Oncogene. 27, 234–243 (2008). 18176605 10.1038/sj.onc.1210908 PMC2821878

[b12] Zhiyu W. . The inflammasome: an emerging therapeutic oncotarget for cancer prevention. *Oncotarget* (2016). 10.18632/oncotarget.9391 PMC5226619 27206676

[b13] Lamkanfi M. & Dixit V. M. Mechanisms and functions of inflammasomes. Cell. 157, 1013–1022 (2014). 24855941 10.1016/j.cell.2014.04.007

[b14] Bruchard M. . Chemotherapy-triggered cathepsin B release in myeloid-derived suppressor cells activates the Nlrp3 inflammasome and promotes tumor growth. Nature medicine. 19, 57–64 (2013). 10.1038/nm.2999 23202296

[b15] Xu Y. . Mycoplasma hyorhinis activates the NLRP3 inflammasome and promotes migration and invasion of gastric cancer cells. PloS one. 8, e77955 (2013). 24223129 10.1371/journal.pone.0077955 PMC3819327

[b16] Wang H. . Upregulation of progranulin by Helicobacter pylori in human gastric epithelial cells via p38MAPK and MEK1/2 signaling pathway: role in epithelial cell proliferation and migration. FEMS immunology and medical microbiology. 63, 82–92 (2011). 21707777 10.1111/j.1574-695X.2011.00833.x

[b17] Labbe K. . M. Cellular inhibitors of apoptosis proteins cIAP1 and cIAP2 are required for efficient caspase-1 activation by the inflammasome. Immunity. 35, 897–907 (2011). 22195745 10.1016/j.immuni.2011.10.016

[b18] Guenthner R. & Vietork L. Surface active materials from perfluorocarboxylic and perfluorosulfonilic acids. I&ED Prod. Res. Dev. 1, 165–169 (1962).

[b19] Shinoda K. & Nomura T. Miscibility of fluorocarbon and hydrocarbon surfactant in micelles and liquid mixtures: basic studies of oil repellent and fire extinguishing agents. J. Phys. Chem. 8, 365–369 (1980).

[b20] Mak Y. . Perfluorinated compounds in tap water from China and several other countries. Environ Sci Technol. 43, 4824–4829 (2009). 19673271 10.1021/es900637a

[b21] Falandysz J. . Is fish a major source of fluorinated surfactants and repellents in humans living on the Baltic Coast? Environmental science & technology. 40, 748–751 (2006). 16509313 10.1021/es051799n

[b22] Houde M. . Biological monitoring of polyfluoroalkyl substances: A review. Environmental science & technology. 40, 3463–3473 (2006). 16786681 10.1021/es052580b

[b23] Kim S. K. & Kannan K. Perfluorinated acids in air, rain, snow, surface runoff, and lakes: relative importance of pathways to contamination of urban lakes. Environmental science & technology. 41, 8328–8334 (2007). 18200859 10.1021/es072107t

[b24] Sinclair E. . Occurrence of perfluoroalkyl surfactants in water, fish, and birds from New York State. Archives of environmental contamination and toxicology. 50, 398–410 (2006). 16435086 10.1007/s00244-005-1188-z

[b25] Calafat A. M. . Serum concentrations of 11 polyfluoroalkyl compounds in the u.s. population: data from the national health and nutrition examination survey (NHANES). Environmental science & technology. 41, 2237–2242 (2007). 17438769 10.1021/es062686m

[b26] Fei C., McLaughlin J. K., Tarone R. E. & Olsen J. Fetal growth indicators and perfluorinated chemicals: a study in the Danish National Birth Cohort. American journal of epidemiology. 168, 66–72 (2008). 18460444 10.1093/aje/kwn095

[b27] Nguyen V. T., Gin K. Y., Reinhard M. & Liu C. Occurrence, fate, and fluxes of perfluorochemicals (PFCs) in an urban catchment: Marina Reservoir, Singapore. Water science and technology: a journal of the International Association on Water Pollution Research. 66, 2439–2446 (2012). 23032776 10.2166/wst.2012.475

[b28] Olsen G. W. . Serum concentrations of perfluorooctanesulfonate and other fluorochemicals in an elderly population from Seattle, Washington. Chemosphere. 54, 1599–1611 (2004). 14675839 10.1016/j.chemosphere.2003.09.025

[b29] Olsen G. W. . Preliminary evidence of a decline in perfluorooctanesulfonate (PFOS) and perfluorooctanoate (PFOA) concentrations in American Red Cross blood donors. Chemosphere. 68, 105–111 (2007). 17267015 10.1016/j.chemosphere.2006.12.031

[b30] Tao L. . Biomonitoring of perfluorochemicals in plasma of New York State personnel responding to the World Trade Center disaster. Environmental science & technology. 42, 3472–3478 (2008). 18522136 10.1021/es8000079

[b31] Olsen G. W. . Half-life of serum elimination of perfluorooctanesulfonate, perfluorohexanesulfonate, and perfluorooctanoate in retired fluorochemical production workers. Environmental health perspectives. 115, 1298–1305 (2007). 17805419 10.1289/ehp.10009 PMC1964923

[b32] Olsen G. W. . Temporal trends of perfluoroalkyl concentrations in American Red Cross adult blood donors, 2000–2010. Environmental science & technology. 46, 6330–6338 (2012). 22554481 10.1021/es300604p

[b33] Brieger A. . Impact of perfluorooctanesulfonate and perfluorooctanoic acid on human peripheral leukocytes. Toxicology in vitro: an international journal published in association with BIBRA. 25, 960–968 (2011). 21397682 10.1016/j.tiv.2011.03.005

[b34] Grandjean P. . Serum vaccine antibody concentrations in children exposed to perfluorinated compounds. Jama. 307, 391–397 (2012). 22274686 10.1001/jama.2011.2034 PMC4402650

[b35] Granum B. . Pre-natal exposure to perfluoroalkyl substances may be associated with altered vaccine antibody levels and immune-related health outcomes in early childhood. Journal of immunotoxicology. 10, 373–379 (2013). 23350954 10.3109/1547691X.2012.755580

[b36] Dong G. H. . Sub-chronic effect of perfluorooctanesulfonate (PFOS) on the balance of type 1 and type 2 cytokine in adult C57BL6 mice. Archives of toxicology. 85, 1235–1244 (2011). 21327619 10.1007/s00204-011-0661-x

[b37] Zheng L. . Type 1 and Type 2 cytokines imbalance in adult male C57BL/6 mice following a 7-day oral exposure to perfluorooctanesulfonate (PFOS). Journal of immunotoxicology. 8, 30–38 (2011). 21299352 10.3109/1547691X.2010.537287

[b38] Wang I. J. . The effect of prenatal perfluorinated chemicals exposures on pediatric atopy. Environmental research. 111, 785–791 (2011). 21601844 10.1016/j.envres.2011.04.006

[b39] Emmett E. A. . Community exposure to perfluorooctanoate: relationships between serum levels and certain health parameters. Journal of occupational and environmental medicine/American College of Occupational and Environmental Medicine. 48, 771–779 (2006). 10.1097/01.jom.0000233380.13087.37 PMC3038254 16902369

[b40] Ryu M. H. . Chronic exposure to perfluorinated compounds: Impact on airway hyperresponsiveness and inflammation. American journal of physiology. Lung cellular and molecular physiology. 307, L765–774 (2014). 25217661 10.1152/ajplung.00100.2014 PMC4233295

[b41] Choi Y. E. . The E3 ubiquitin ligase cIAP1 binds and ubiquitinates caspase-3 and -7 via unique mechanisms at distinct steps in their processing. The Journal of biological chemistry. 284, 12772–12782 (2009). 19258326 10.1074/jbc.M807550200 PMC2676007

[b42] Eckelman B. P., Salvesen G. S. & Scott F. L. Human inhibitor of apoptosis proteins: why XIAP is the black sheep of the family. EMBO reports. 7, 988–994 (2006). 17016456 10.1038/sj.embor.7400795 PMC1618369

[b43] Bertrand M. J. . Cellular inhibitors of apoptosis cIAP1 and cIAP2 are required for innate immunity signaling by the pattern recognition receptors NOD1 and NOD2. Immunity. 30, 789–801 (2009). 19464198 10.1016/j.immuni.2009.04.011

[b44] Tseng P. H. . Different modes of ubiquitination of the adaptor TRAF3 selectively activate the expression of type I interferons and proinflammatory cytokines. Nature immunology. 11, 70–75 (2010). 19898473 10.1038/ni.1819 PMC2872790

[b45] Conte D. . Inhibitor of apoptosis protein cIAP2 is essential for lipopolysaccharide-induced macrophage survival. Molecular and cellular biology. 26, 699–708 (2006). 16382159 10.1128/MCB.26.2.699-708.2006 PMC1346893

[b46] Dagenais M. . critical role for cellular inhibitor of protein 2 (cIAP2) in colitis-associated colorectal cancer and intestinal homeostasis mediated by the inflammasome and survival pathways. Mucosal Immunology. 9, 146–158 (2016). 26037070 10.1038/mi.2015.46

[b47] Mayer B. . Inhibitor of Apoptosis Proteins as Novel Targets in Inflammatory Processes. Arterioscler Thromb Vasc Biol. 31, 2240–2250 (2011). 21817100 10.1161/ATVBAHA.111.234294

[b48] Basseres D. S. & Baldwin A. S. Nuclear factor-kappaB and inhibitor of kappaB kinase pathways in oncogenic initiation and progression. Oncogene. 25, 6817–6830 (2006). 17072330 10.1038/sj.onc.1209942

[b49] Karin M. Nuclear factor-kappaB in cancer development and progression. Nature. 441, 431–436 (2006). 16724054 10.1038/nature04870

[b50] Hacker H. & Karin M. Regulation and function of IKK and IKK-related kinases. *Science’s STKE: signal transduction knowledge environment.* re13 (2006). 10.1126/stke.3572006re13 17047224

[b51] Gross O., Thomas C. J., Guarda G. & Tschopp J. The inflammasome: an integrated view. Immunological reviews. 243, 136–151 (2011). 21884173 10.1111/j.1600-065X.2011.01046.x

[b52] Hoesel B. & Schmid J. A. The complexity of NF-kappaB signaling in inflammation and cancer. Molecular cancer. 12, 86 (2013). 23915189 10.1186/1476-4598-12-86 PMC3750319

[b53] Rathinam V. A., Vanaja S. K. & Fitzgerald K. A. Regulation of inflammasome signaling. Nature immunology. 13, 333–342 (2012). 22430786 10.1038/ni.2237 PMC3523703

[b54] Qiao Y. . TLR-induced NF-kappaB activation regulates NLRP3 expression in murine macrophages. FEBS letters. 586, 1022–1026 (2012). 22569257 10.1016/j.febslet.2012.02.045

[b55] Liu M. H. FGF-21 alleviates diabetes-associated vascular complications: Inhibiting NF-kappaB/NLRP3 inflammasome-mediated inflammation? International journal of cardiology. 185, 320–321 (2015). 25828673 10.1016/j.ijcard.2015.03.165

[b56] Xiang P. . NZ suppresses TLR4/NF-kappaB signalings and NLRP3 inflammasome activation in LPS-induced RAW264.7 macrophages. Inflammation research: official journal of the European Histamine Research Society. 64, 799–808 (2015). 10.1007/s00011-015-0863-4 26298161

[b57] Bertrand M. J. . cIAP1 and cIAP2 facilitate cancer cell survival by functioning as E3 ligases that promote RIP1 ubiquitination. Molecular cell. 30, 689–700 (2008). 18570872 10.1016/j.molcel.2008.05.014

[b58] Varfolomeev E. . IAP antagonists induce autoubiquitination of c-IAPs, NF-kappaB activation, and TNFalpha-dependent apoptosis. Cell. 131, 669–681 (2007). 18022362 10.1016/j.cell.2007.10.030

[b59] Vince J. E. . IAP antagonists target cIAP1 to induce TNFalpha-dependent apoptosis. Cell. 131, 682–693 (2007). 18022363 10.1016/j.cell.2007.10.037

